# Who gets prescriptions for proton pump inhibitors and why? A drug-utilization study with claims data in Bavaria, Germany, 2010–2018

**DOI:** 10.1007/s00228-021-03257-z

**Published:** 2021-12-08

**Authors:** Ina-Maria Rückert-Eheberg, Michael Nolde, Nayeon Ahn, Martin Tauscher, Roman Gerlach, Florian Güntner, Alexander Günter, Christa Meisinger, Jakob Linseisen, Ute Amann, Sebastian-Edgar Baumeister

**Affiliations:** 1 Institute for Medical Information Processing, Biometry, and Epidemiology (IBE), Ludwig-Maximilians-University Munich, Marchioninistr. 15, 81377 Munich, Germany; 2 Chair of Epidemiology, University of Augsburg at University Hospital Augsburg, Stenglinstr. 2, 86156 Augsburg, Germany; 3 Institute of Epidemiology, Helmholtz Zentrum München, German Research Center for Environmental Health (GmbH), Ingolstädter Landstr. 1, 85764 Neuherberg, Germany; 4 Association of Statutory Health Insurance Physicians in Bavaria (Kassenärztliche Vereinigung Bayerns, KVB), Elsenheimerstr. 39, 80687 Munich, Germany; 5 AOK Bayern - Die Gesundheitskasse, Carl-Wery-Str. 28, 81739 Munich, Germany; 6 Independent Research Group Clinical Epidemiology, Helmholtz Zentrum München, German Research Center for Environmental Health (GmbH), Ingolstädter Landstr. 1, 85764 Neuherberg, Germany; 7 Institute of Health Services Research in Dentistry, University of Münster, Albert-Schweitzer-Campus 1, 48149 Münster, Germany

**Keywords:** Acid-related diseases, Drug use, Indications, Proton pump inhibitors

## Abstract

**Purpose:**

The German annual drug prescription-report has indicated overuse of proton pump inhibitors (PPIs) for many years; however, little was known about the characteristics of people using PPIs. This study aimed to provide comprehensive utilization data and describe frequencies of potential on- and off-label PPI-indications in Bavaria, Germany.

**Methods:**

Claims data of statutorily insured people from 2010 to 2018 were used. Defined daily doses (DDDs) of PPIs by type of drug, prevalence of PPI-use and DDDs prescribed per 1000 insured people/day were analyzed. For 2018, proportions of users and DDDs per 1000 insured people were calculated by age and sex. To elucidate changes in prescribing practices due to a suspected drug-drug interaction, we examined co-prescribing of clopidogrel and PPIs between 2010 and 2018. For PPI new users, sums of DDDs and frequencies of potential indications were examined.

**Results:**

PPI prescribing increased linearly from 2010 to 2016 and gradually decreased from 2016 to 2018. In 2018, 14.7% of women and 12.2% of men received at least one prescription, and 64.8 DDDs (WHO-def.) per 1000 insured people/day were prescribed. Overall, omeprazole use decreased over the observation period and was steadily replaced by pantoprazole, especially when co-prescibed with clopidogrel. An on-label PPI-indication was not reported at first intake in 52.0% of new users.

**Conclusions:**

The utilization of prescribed PPIs has decreased since 2016. However, a large proportion of new PPI-users had no documentation of a potential indication, and the sums of DDDs prescribed often seemed not to comply with guidelines.

**Supplementary Information:**

The online version contains supplementary material available at 10.1007/s00228-021-03257-z.

## Introduction

Almost like a pandemic, proton pump inhibitors (PPIs) have spread around the globe and are labelled “lifestyle medications” not without reason. They were introduced to the German market in 1989 and have been used as prescription and over-the-counter drugs (since 2009) to treat heartburn, gastroesophageal reflux disease (GERD) and peptic ulcers, to eradicate the stomach pathogen *Helicobacter pylori* in combination with antibiotics, and to address off-label gastrointestinal complaints [1, 2]. In recent years, associations of PPI intake with various diseases (e.g., bacterial infections, pneumonia, kidney disease, cardiovascular diseases, and dementia [3]) have emerged. The validity and clinical relevance of these observations is unresolved. Despite such reports, several international studies [4–8] described increased use, and longer intake of PPIs than recommended by guidelines, often without appropriate clinical indications. For Germany, trends in PPI utilization and associated costs are regularly published by the German annual drug prescription-report [9]. However, these analyses do not elaborate on differences by sex and age, duration of intake, frequencies of indications, or other characteristics. A study using data of one German statutory health insurance company from 2005 to 2013 found that the PPI prescribing prevalence increased from 8.2 to 16.2% during this period, women used PPIs more often than men, and elderly people were affected much more frequently than children and younger adults [10]. However, to the best of our knowledge, no further comprehensive utilization study in Germany has been conducted. Moreover, PPIs (in particular omeprazole) have been suspected to reduce the effectiveness of the antiplatelet agent clopidogrel, because both drugs use the same metabolizing pathway featuring the cytochrome P450 enzyme CYP2C19 (e.g., [11]). In consequence, the European Medicines Agency (EMA) issued a safety warning in 2009—updated in 2010—and discouraged conjoint use of clopidogrel and omeprazole or esomeprazole [12]. Thus, the present analysis used claims data of all statutorily insured people in Bavaria, to describe trends in PPI-use from 2010 to 2018, to characterize utilization by sex and age, to examine if clopidogrel was prescribed less often together with omeprazole/esomeprazole, and to elucidate clinical PPI on- and off-label indications.

## Methods

### Data source and participants

In Germany, health insurance has been compulsory since 2009. It consists of two systems: the statutory health insurance (SHI) and the private health insurance sectors. Depending on their type of employment, income, or earlier type of insurance, people can choose their insurance scheme [13]. Only a small proportion of inhabitants in Bavaria (12.7% in 2016 [14]) is insured by private health insurance companies. According to self-assessment and objective data, this part of the population is somewhat healthier and more prosperous on average [15]. The current analysis did not include data of privately insured people and is thus only representative of the SHI population of Bavaria.

In the SHI sector, the KVB (German, Kassenärztliche Vereinigung Bayerns; English, Association of Statutory Health Insurance Physicians of Bavaria) is the largest of 17 regional associations of statutory health insurance physicians in Germany; represents about 26,000 physicians and psychotherapists towards politics, health insurance companies, and the public; makes sure that every SHI-insured patient has access to medical care at all times; and negotiates contracts with associations of SHI funds. It is subject to the legal supervision of the Bavarian State Ministry of Health and Care [16].

For this study, the KVB provided an anonymized dataset of outpatient claims data from all SHI companies in the Federal State of Bavaria, encompassing the calendar years 2010 to 2018. The data included information on patients’ year of birth, sex, codes according to the Anatomical Therapeutic Chemical Classification (ATC) of prescription medications (excluding medications that were prescribed but not reimbursed by SHI; no information on over-the-counter medications was available) with prescription dates, defined daily doses (DDDs) and pharmacy registration numbers (Pharmazentralnummern, PZN), diagnoses (coded by the International Classification of Diseases (ICD)-10 system), medical treatments and assessments including dates (coded by the German “Einheitlicher Bewertungsmaßstab” (EBM) system), and physician specialities. Diagnoses were transferred by physicians on a quarterly basis without exact dates, while data on reimbursable medications were acquired from pharmacies. Inpatient claims data are generally not registered by the KVB. All age groups were included. The Ethics committee at the Ludwig Maximilians-University of Munich confirmed that an ethics approval was not needed since only anonymized claims data were used. For the same reason, informed consent by the insured individuals was not required.

### Definition of PPIs and defined daily doses

During the study period, six PPIs as single-agent products and two combination products used to eradicate *Helicobacter pylori* were dispensed in Bavaria (Table 1). Except for omeprazole and dexlansoprazole, the German definitions [17] of DDDs differ from those of the World Health Organization (WHO) [18], since in 2005, the German Ministry of Health decided to adapt the DDDs according to the doses recommended in long-term treatment of GERD, the main indication in Germany [19]. To enable comparisons with other German analyses and international studies, we used both definitions and indicated which one was used in the respective legends.

**Table 1:** Proton pump inhibitors prescribed and dispensed in Bavaria (2010–2018)

Drug	ATC	German-DDD (mg)	WHO-DDD (mg)	Available doses of PPI per unit (mg)
Omeprazole	A02BC01	20 O^†^, P^‡^	20 O, P	10, 20, 40
Pantoprazole	A02BC02	20 O, P	40 O, P	20, 40
Lansoprazole	A02BC03	15 O	30 O	15, 30
Rabeprazole	A02BC04	10 O, P	20 O, P	10, 20
Esomeprazole	A02BC05	20 O, P	30 O	10, 20, 40
Dexlansoprazole (since 2014)	A02BC06	30 O	30 O	30, 60
**Combinations to eradicate *Helicobacter pylori***				
Omeprazole (with Amoxicillin and Clarithromycin)	A02BD05 (since 2012)	Six dose units incl. 14 DDD Omeprazole	Six dose units incl. 14 DDD Omeprazole	20
Pantoprazole (with Amoxicillin and Clarithromycin)	A02BD04	Six dose units incl. 28 DDD Pantoprazole	Six dose units incl. 14 DDD Pantoprazole	40

*O*^*†*^ oral, *P*^*‡*^ parenteral

### Definition of variables and analyses performed

For the initial analysis (1), we calculated the total amounts of PPIs prescribed by summing up DDDs per active agent for the years 2010 to 2018 (German DDD-definitions). DDDs from combination products used to erase *Helicobacter pylori* were converted and added to the amounts of pantoprazole or omeprazole, respectively.

Next (2), we assessed the annual prevalence of PPI-use (at least one prescription) per 100 insured persons [20]. The prevalence was stratified by sex and standardized to the age distribution of the population of SHI-insured people in Bavaria [20] in 2010. For 2018, the most current year in our dataset, the sex-specific raw prevalence by 5-year-age groups was calculated.

To enable international comparisons (3), DDDs (WHO-definition) prescribed per 1000 insured people per day were computed across all observation years. Additionally, for 2018, DDDs per 1000 insured people by type of PPI, sex and age-groups were calculated.

Changes in co-prescribing of PPIs and histamine H2-receptor antagonists (H2RAs, ATC code: A02BA) in people who used clopidogrel (ATC codes: B01AC04, B01AC34) (4) were analyzed from 2010 to 2018. We hypothesized that the safety warning issued regarding the clopidogrel-omeprazol interaction might have resulted in a fallback on prescription of H2RAs. Since PPI- and H2RA-packages with 100 tablets are available and daily intake is not necessarily required (i.e., the package could be used for longer periods than 100 days including breaks), we defined concomitant use as at least one prescription of a PPI/H2RA one-quarter before, in the same quarter, or one-quarter after the quarter that included a prescription for clopidogrel. The prevention of clopidogrel-induced bleeding may thus have been the indication for PPI/H2RA use, but other indications could also have been present before, during, or after the clopidogrel prescription date. Since data from the fourth quarter of 2009 and the first quarter of 2019 were not available, Fig. 4 covers the time period between the second quarter of 2010 and the third quarter of 2018, inclusively.

A cohort of new PPI-users was selected (5) (no PPI prescription during 2016, initiation of use in 2017, and follow-up until the end of 2018 at the latest; these years were chosen since the most current data available in the dataset were from 2018, and we intended to capture at least 12 months of possible PPI intake for each user) and described regarding the sum of prescribed PPI-DDDs to estimate the duration of intake, long-term use (> 180 DDDs during the observation period, analogous to a theoretical daily intake of 6 months), clinical indication for PPI prescription or lack thereof, intake of ulcerogenic co-medications, and esophagogastroduodenoscopy (defined by EBM-numbers: 13400, 04,511, 04,515) performed during 2017 or 2018 in long-term users. Diagnoses for on- and off-label use were taken into account, if they had been reported one-quarter before, in the same, or one-quarter after the first PPI prescription. On-label indications according to the Summary of Product Characteristics [21] and package information were heartburn (R12), GERD (K21), ulcer (K25-K28), infection with *Helicobacter pylori* (B98), esophagitis (K20), and Zollinger-Ellison syndrome (K16.4). In users who had no documentation of an on-label indication, we regarded gastritis and duodenitis (K29), dyspepsia (K30), upper stomach pain/unspecified dyspepsia (R10.1), hernia diaphragmatica (K44), other diseases of the stomach or duodenum (K31), other diseases of the digestive system including bleeding (K92), other diseases of the esophagus (K22, K23), Crohn’s disease (K50), and cancer of the esophagus or stomach (C15, C16) as potential off-label indications. Ulcerogenic co-medications were considered if they had been prescribed on the same day as the PPI or up to 90 days before this date. Guideline recommendations (modified according to Fischbach [2, 22], Table 2) were used to assess the appropriateness of PPI-use due to co-medications: In people aged 65 years or older, monotherapy with a non-steroidal anti-inflammatory drug, acetylsalicylic acid (as antiplatelet agent), a direct oral anticoagulant, a vitamin K-antagonist, or a combination of at least two other types of anti-thrombotic medications justify PPI-use. In people younger than 65 years, polypharmacy with different drug combinations (including dual platelet inhibition) may require gastro-protection with a PPI. The exact length of the observation period for each incident PPI-user could not be calculated, since information on drop-out from SHI or death were not available.

**Table 2:** Characteristics of incident PPI-users

Age groups (years)	0–19		20–34		35–49		50–64		65–79		80 +		Total
***N* (%)**	**21,210** **(3.7)**		**85,243** **(14.9)**		**120,557** **(21.1)**		**166,944** **(29.2)**		**121,737** **(21.3)**		**56,233** **(9.8)**		**571,924** **(100.0)**
Women (%)	57.6		56.1		56.8		56.1		58.3		64.2		57.6
German-DDD: median (25th percentile, 75th percentile)	30 (28, 74)		60 (30, 120)		60 (30, 180)		100 (58, 210)		130 (60, 336)		200 (90, 508)		100 (56, 208)
WHO-DDD: median (25th percentile, 75th percentile)	30 (14, 55)		30 (15, 60)		45 (28, 100)		60 (30, 120)		86 (30, 200)		100 (50, 280)		60 (30, 120)
Long-term users (German-DDD > 180) (%)	8.7		16.0		25.6		34.3		46.3		57.3		33.6
Long-term users (WHO-DDD > 180) (%)	4.1		6.3		12.0		18.3		27.4		37.6		18.5
**PPI on-label indications (previous, same, or one quarter after first PPI prescription)**													
Gastroesophageal reflux disease (GERD) (K21) (%)	10.9	16.8		21.3		23.9		24.7		18.5		21.5	
Heartburn (R12) (%)	2.9	4.8		4.1		3.5		2.7		1.6		3.4	
Peptic ulcer (K25-K28) (%)	0.7	1.5		1.9		2.4		3.1		3.6		2.4	
*Helicobacter pylori* (B98) (%)	1.4	2.2		2.8		2.6		2.1		1.0		2.3	
Esophagitis (K20) (%)	0.6	0.6		0.7		0.7		0.7		0.5		0.6	
Zollinger-Ellison syndrome (E16.4) (%)	0.00	0.01		0.01		0.02		0.01		0.01		0.01	
**Co-medications (up to 90 days before or at same day as first PPI prescription)**													
NSAID^‡^ (M01A, M01B, N02A, N02B, C10BX) (%)	35.3	39.8		48.1		51.1		48.9		55.8		48.2	
Antithrombotic drugs (B01A) (%)	5.4	5.0		5.7		11.0		23.7		37.1		14.0	
Corticosteroids (H02) (%)	3.7	5.4		7.6		8.5		9.7		9.6		8.0	
SSRI^§^ (N06AB) (%)	1.0	2.1		3.3		3.9		3.6		5.1		3.5	
**PPIs possibly used to prevent medication-related ulceration (on-label indication)† (%)**	6.6	7.6		10.1		14.4		57.9		69.2		26.8	
**Proportion without on-label PPI-indication (including†) at first intake (%)**	78.8	70.7		64.9		59.7		25.0		21.3		52.0	
**Proportion without PPI on-label (including†) or off-label indication at first intake (%)**	42.9	41.7		43.8		42.9		16.6		15.4		34.6	
**Off-label indications (previous, same, or one quarter after first PPI prescription) in users without documentation of an on-label indication**													
**Age groups (years)**	**0–19**	**20–34**		**35–49**		**50–64**		**65–79**		**80 + **		**Total**	
**N (%)**	**16,702** **(5.6)**	**60,258** **(20.3)**		**78,279** **(26.3)**		**99,706** **(33.5)**		**30,397** **(10.2)**		**11,979** **(4.0)**		**297,321** **(100.0)**	
Gastritis/duodenitis (K29) (%)	34.5	30.4		23.6		20.1		23.0		18.0		24.1	
Upper stomach pain/dyspepsia, not specified (R10.1) (%)	13.0	12.3		9.4		6.8		6.9		4.5		8.8	
Hernia diaphragmatica (K44) (%)	0.4	1.0		1.6		2.4		4.6		3.7		2.1	
Other diseases of stomach/duodenum (K31) (%)	2.1	2.2		2.0		1.8		2.3		1.7		2.0	
Dyspepsia (K30) (%)	1.8	2.0		1.5		1.3		1.4		1.3		1.5	
Other diseases of the digestive system incl. bleeding, (K92) (%)	1.5	1.4		1.1		1.3		2.0		2.8		1.4	
Other diseases of esophagus (K22, K23) (%)	0.2	0.3		0.4		0.8		1.8		1.5		0.7	
Crohn’s disease (K50) (%)	0.7	1.1		0.8		0.6		0.5		0.2		0.7	
Cancer of esophagus/stomach (C15, C16) (%)	0.0	0.0		0.1		0.3		0.8		1.1		0.2	

**need for gastro-protection modified according to Fischbach, 2019:**

-age >  = 65 years: monotherapy with NSAID OR ASA (as anti-thrombotic agent, ATC = B01AC06) OR DOAC OR VKA OR combination of at least two other types of antithrombotic medication

-age < 65 years: prescription of NSAID together with anti-thrombotic medication or corticosteroid or SSRI or diagnosis of ulcer; prescription of ASA OR DOAC OR VKA together with corticosteroid or SSRI or diagnosis of ulcer; prescription of a combination of different classes of anti-thrombotic medications (i.e. Heparin and DOAC) or dual platelet inhibition (ASA with clopidogrel, prasugrel or ticagrelor)

*NSAID* non-steroidal anti-inflammatory drug, *SSRI *selective serotonin reuptake inhibitor, *ASA* acetylsalicylic acid, *DOAC* direct oral anticoagulant, *VKA* vitamin K-antagonist

All analyses were carried out using R (version 3.6.3).

## Results

**Fig. 1:**
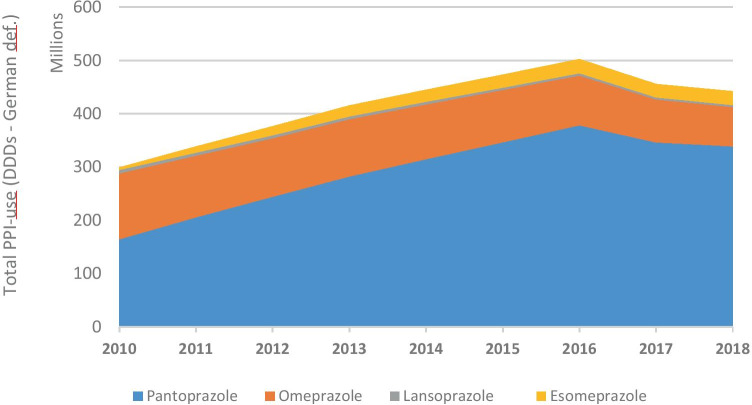
Dispensed DDDs (German definition) of proton pump inhibitors per calendar year during the study period, stratified by active agent (dexlansoprazole and rabeprazole not shown due to small numbers, all age groups)

**Fig. 2:**
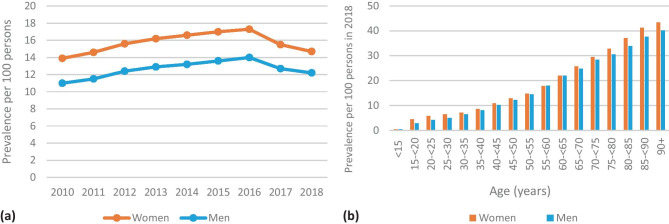
(**a**) Prevalence of PPI-use (at least one prescription) per 100 insured people, 2010–2018, age-standardized to 2010. (**b**) Age-and sex-specific prevalence of PPI-use (at least one prescription) per 100 insured people in 2018 (unstandardized)

**Fig. 3:**
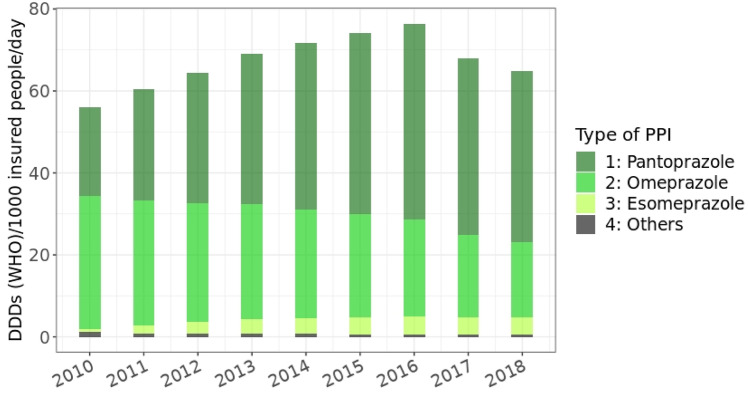
DDDs (WHO) per 1000 insured people per day, 2010–2018

**Fig. 4:**
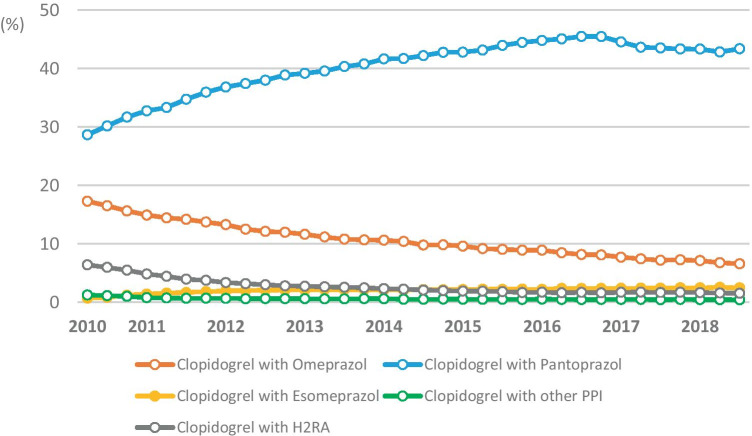
Proportions of co-prescriptions of clopidogrel and different PPIs/H2RA in people who used clopidogrel, quarterly assessment, 2010/quarter 2 to 2018/quarter 3

1. *Total amounts of PPIs prescribed during the years 2010–2018*
  The number of SHI-insured people increased from 10.4 million in 2010 to 11.1 million in 2018, and the proportions of the age groups shifted slightly (e.g., decrease in age group 0–19 years from 19.3% in 2010 to 17.8% in 2018, increase in people aged 75 and older from 9.5% in 2010 to 11.0% in 2018) [20]. The total amount of prescribed DDDs increased steadily from 301.2 million DDDs in 2010 to 503.2 million DDDs in 2016 and then declined until the end of the observation period (Fig. 1). Use of pantoprazole accounted for the largest share, followed by omeprazole, esomeprazole, and lansoprazole. Only small amounts of rabeprazole (0.9 million DDDs in 2018) and dexlansoprazole (112 DDDs in 2018) were used (not shown in Fig. 1). In 2018, 71.2% of DDDs was prescribed by primary care physicians (315.3 million DDDs), followed by internal medicine specialists (119.1 million DDDs, 26.9%) and other physician specialties (8.7 million DDDs, 1.9%).
2. *Prevalence of PPI-use per 100 insured people*
  The age-standardized proportion of PPI-users per 100 insured people increased in line with the total amount used until 2016 and declined afterwards (Fig. 2a). In 2018, 14.7% of women and 12.2% of men received at least one PPI prescription. The stratified figure shows that PPI-use depended on age: While 0.4% of the boys and 0.5% of the girls under 15 years of age received at least one prescription for PPIs in 2018, this proportion reached 40.2% and 43.4% in men and women aged 90 years and older, respectively. Women of all age groups were more often PPI-users than men (Fig. 2b).
3. *DDDs prescribed per 1000 insured people per day*
  In 2010, 56.1 DDDs (based on the WHO-definition) of PPIs per 1000 insured people per day were dispended. The number increased to 76.4 DDDs per 1000 insured per day in 2016 and decreased to 64.8 in 2018 (Fig. 3). Figure S1 shows DDDs per 1000 insured people in 2018 stratified by sex, age-groups, and types of PPI. In people younger than 20 years, the greatest amount of DDDs prescribed was due to omeprazole (56.3% of DDDs per 1000 insured in men, 50.0% per 1000 insured in women), followed by pantoprazole. In all other age groups, pantoprazole made up more than 60% of DDDs per 1000 insured people.
4. *Changes in co-prescribing of clopidogrel with PPIs*
  Clopidogrel was the predominantly prescribed antiplatelet drug by far in 2018 (81.3% of users of clopidogrel, ticagrelor, or prasugrel received at least one prescription; ticagrelor, 14.0%; prasugrel, 6.8%). However, omeprazole was co-prescribed to only 6.6% of clopidogrel users in 2018/third quarter, while this proportion had been 16.5% in 2010/third quarter. Esomeprazole was used by 0.8% of patients with clopidogrel treatment in 2010/third quarter, and the proportion increased to 2.5% in 2018/third quarter. The proportion of conjoint use of clopidogrel and pantoprazole increased from 30.2% in 2010/third quarter to 43.4% in 2018/third quarter, while co-prescriptions with H2RAs decreased from 6.0% in 2010/third quarter to 1.5% in 2018/third quarter (Fig. 4). The co-prescribing patterns of PPIs with ticagrelor and prasugrel were very similar: In particular, concomitant use with omeprazole decreased clearly and approximately half of the patients used pantoprazole (data not shown). Other PPIs and H2RAs played minor roles in the treatment of patients who took antiplatelet drugs.
5. *Characteristics of new PPI-users*
  We identified 571,924 subjects who had not received a prescription for PPIs in 2016 and started using them in 2017 (Table 2). The median of the total amount of DDDs prescribed in 2017 and 2018 was 30 DDDs in people younger than 20 years and 200 DDDs in people older than 79 years. A 33.6% of all incident PPI-users received prescriptions that provided a standard dose of PPIs daily for more than 6 months. The most common on-label indication was GERD (21.5%), followed by heartburn (3.4%), ulcer (2.4%), and infection with *Helicobacter pylori* (2.3%). Esophagitis and Zollinger Ellison-syndrome were very rare (0.6% and 0.01%) (Table 2). In users who had no documentation of an on-label indication (*N* = 297,321), gastritis/duodenitis (24.1%) and upper stomach pain/unspecified dyspepsia (8.8%) were most frequently reported, while other potential off-label indications were found in 2% or less of the users. A 26.8% of PPI-users had co-medications and further risk factors that may warrant prevention of medication-related ulceration. Thus, an appropriate on-label indication (including gastro-protection) was not documented in 52.0% of incident PPI-users. For 34.6% of the incident users, neither an on-label nor an off-label PPI-indication could be identified around their first PPI prescription. The proportion was 42.9% in the youngest age group and 15.4% in the elderly, 80 years and older. Furthermore, 27.0% of the long-term users were examined by gastro-intestinal endoscopy.

## Discussion

Our claims data analysis showed that the reimbursement by SHI of PPIs in Bavaria increased steadily from 2010 to 2016 despite the option to buy PPIs over the counter during the observational period. The number of PPIs prescribed in Bavaria accounted for 13% of the amount prescribed throughout Germany [9], while the SHI statutorily insured population of Bavaria made up 15% of the SHI population of Germany. The decrease in 2017 and 2018 may be due to the growing attention to possible adverse effects. Furthermore, in December 2016, a new quantity target was implemented in medical practices to reduce PPI prescriptions per prescription case by 10% [23]. Drug targets are part of the annually negotiated framework for the control of pharmaceutical expenditures between the KVB and SHI companies in Bavaria and are based on clinical and economic deliberations.

Different types of PPIs are preferred in different countries even though none of the agents appears to have greater efficiency than the others at equivalent dosing, and their pharmacological and pharmacokinetic properties are very similar [24]. Pantoprazole was prescribed most often in Bavaria, while omeprazole and others were preferred in many other countries, e.g., Iceland [4] (2003–2015, omeprazole, esomeprazole, and rabeprazole), the UK [25] (1990–2018, omeprazole and lansoprazole), France [5] (2015, omeprazole), Denmark [26] (2014, omeprazole, pantoprazole, and lansoprazole in roughly equal parts), and Australia [27] (2017, esomeprazole followed by pantoprazole). These differences are due to different healthcare systems, drug approval processes, pharma contracts, and probably also due to differences in lobby work at physician’s offices and pharmacies.

The additional relative reduction in the dispensing of omeprazole in Bavaria was probably due to the growing awareness of the suspected decrease in efficacy of the antiplatelet agent clopidogrel induced by omeprazole/esomeprazole [28] and the EMA safety communication. Conjoint use of clopidogrel and esomeprazole increased during this time, but the absolute proportions were very small. In people who received prescriptions of other antiplatelet drugs such as ticagrelor and prasugrel, omeprazole was also co-prescribed more infrequently (data not shown), and pantoprazole was used most often. In the USA, the Food and Drug Administration (FDA) also issued safety warnings, whereupon the prevalence of concomitant treatment of clopidogrel and PPIs in patients with acute coronary syndrome decreased clearly. In 2016, the prevalence of concomitant use of clopidogrel with omeprazole or esomeprazole was down to 0.8% (while it was 18.4% in 2008), and, contrary to our findings, use of H2RAs increased temporarily from 2009 to 2011 [29].

Treatment guidelines in Germany recommend short-term treatment of 4 to 8 weeks (and if necessary, a continuation with low-dose on-demand treatment) for most patients with gastroesophageal reflux, non-erosive esophagitis, and peptic ulcers [1]. Accordingly, long-term treatment should only be required in rare cases with chronic hypersecretion, such as in Zollinger-Ellison syndrome, complicated ulcerations, gastric bleedings, severe GERD, and for gastro-protection, especially in patients aged 65 years and older [30, 31]. About one-third of all incident PPI-users received prescriptions that in sum provided a standard dose of PPIs daily for more than 6 months. Particularly in the younger age groups (< 65 years), this observation cannot be explained by the frequencies of serious chronic PPI-indications and therefore, to a large extent, does not seem to comply with the guidelines. Moreover, in more than 40% of incident users younger than 65 years, no plausible on- or off-label indication was reported, whatsoever. It is important to note, though, that heartburn may be underreported systematically since it is not considered a serious condition.

In studies around the world (e.g., [4–6, 26, 32–35]), similar trends and proportions of unreasonable PPI-use have been reported. The amounts used per 1000 people differed by country, by age group, and by calendar year; however, the developments of the drug use were comparable. Naunton et al. [7] summarized 34 international studies from 2000 to 2016 and concluded that the extent of unreasonable prescribing in hospital and community settings may be about 50% and did not improve in recent years. In Iceland [4], the prevalence of PPI-prescribing in people aged 19 years and older increased from 8.5% in 2003 to 15.5% in 2015. A study from Denmark [26] found that the number of adult PPI-users increased fourfold from 2002 to 2014. In France, almost 30% of the adult population used PPIs in 2015 and 32.4% of the new users lacked a rational indication or co-medication [5]. A comparison of data from Australia and South Korea reported that the number of DDDs prescribed per 1000 population per day in Australia increased from about 50 in 2004 to more than 70 in 2015 and from almost 0 in 2004 in South Korea to more than 20 in 2017 [27]. All studies inferred that rational use should be promoted and physicians need to be guided in de-prescribing PPIs. Often, lifestyle modifications, e.g., smoking cessation, weight loss, healthy nutrition [36], and stress reduction [37], could likely improve acid-related symptoms and decrease long-term intake of PPIs; however, the process of weaning should be discussed thoroughly by the patient and his physician and taken step by step in order to avoid failure, e.g., due to acid-rebound hypersecretion [38]. Though evidence is still scarce, some studies have shown that discontinuing the intake or tapering the dose of PPIs can be successful in many patients [38–40] and de-prescribing guidelines have been developed (e.g., [41–44]).

## Strengths and limitations

Claims data analyses are distinguished by large, unselected, and relatively current population-based datasets that allow the follow-up of individual persons over time. Some types of bias in observational studies, e.g., non-response and recall bias, are not an issue. In our analysis, we considered out-patient data of all statutorily insured people in Bavaria over a period of 9 years. In contrast to many other claims data studies, our dataset included diagnoses and prescription data. Lack of clinical data, missing information on lifestyle factors, and the unclear validity of diagnoses are some limitations. Moreover, medications purchased over the counter and medications that were prescribed but not reimbursed by SHI are not included. We may have overestimated the actual consumption of PPIs since we could not ascertain patient compliance. Moreover, physicians may tend to prescribe large packages for economic reasons and instruct their patients to use the tablets on-demand until the expiry date has been reached. This practice could lead to a significant dumping of PPIs. Generally, prescription and diagnosing practices can be distorted by financial incentives and change according to new framework conditions of the audit agreements imposed by regulatory authorities.

### Conclusions

As elsewhere, in Bavaria, the utilization of PPIs has reached an extent that cannot be explained by an equally sharp rise of underlying conditions. Our study found that for more than half of new PPI-users, no potential on-label indication could be identified, and about one-third had no record of any likely on- or off-label indication. Dispensing of omeprazole decreased overproportionately, which was probably due to growing awareness of its suspected interaction with the anticoagulant agent clopidogrel via the cytochrome P450 pathway. In recent years, a moderate overall reduction of PPI utilization has taken place in Bavaria, a development that, inter alia, may be due to a new PPI quantity target imposed by the Bavarian regulatory authorities at the end of 2016.

## Supplementary Information

Below is the link to the electronic supplementary material.

Supplementary file1 (DOCX 158 KB)

## Data Availability

The data that support the findings of this study are available from the Bavarian Association of Statutory Health Insurance Physicians (German: Kassenärztliche Vereinigung Bayerns, KVB) by contractual agreement.

## Abbreviations

ASA: Acetylsalicylic acid
ATC: Anatomical Therapeutic Chemical Classification System
DDD: Defined daily dose
DOAC: Direct oral anticoagulant
EBM: Einheitlicher Bewertungsmaßstab (German Physician Fee schedule)
EMA: European Medicines Agency
GERD: Gastroesophageal reflux disease
H2RA: Histamine H2-receptor antagonist
ICD: International Classification of Diseases
NSAID: Nonsteroidal anti-inflammatory drug
PPI: Proton pump inhibitor
SHI: Statutory health insurance
SSRI: Selective serotonin reuptake inhibitor
VKA: Vitamin K-antagonist

## Code availability

All analyses were carried out using R (version 3.6.3).
