# Supplementary material for: Who gets prescriptions for proton pump inhibitors and why? A drug-utilization study with claims data in Bavaria, Germany, 2010–2018
Source: Eur J Clin Pharmacol. 2021 Dec 8;78(4):657–67. doi: 10.1007/s00228-021-03257-z (PMC8927002; doi:10.1007/s00228-021-03257-z)
Supplement: Supplementary file 1 — Supplementary file1 (DOCX 158 KB) [file 228_2021_3257_MOESM1_ESM.docx]

Supplement

**Who gets prescriptions for proton pump inhibitors and why? A drug-utilization study with claims data in Bavaria, Germany, 2010-2018**

European Journal of Clinical Pharmacology

Ina-Maria Rückert-Eheberg, Michael Nolde, Nayeon Ahn, Martin Tauscher, Roman Gerlach, Florian Güntner, Alexander Günter, Christa Meisinger, Jakob Linseisen, Ute Amann, Sebastian-Edgar Baumeister

**Corresponding author**

Dr. Ina-Maria Rückert-Eheberg

Chair of Epidemiology, University of Augsburg at University Hospital Augsburg / Ludwig-Maximilians-University Munich

Stenglinstr. 2, 86156 Augsburg

Email: ina-maria.rueckert@helmholtz-muenchen.de

Tel: +49 89 3187 49481

ORCID: 0000-0001-5418-283X

**
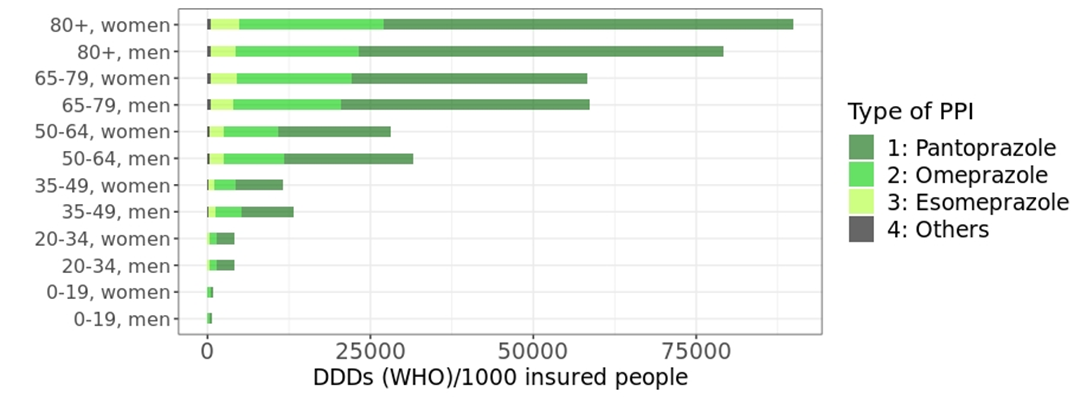
**

**Supplementary data. Fig. S1.** Age-, sex- and agent-specific DDDs (WHO) used per 1000 insured people in 2018
